# Beyond *CCR5* and *HLA*: rare genetic variants in HIV acquisition and disease progression

**DOI:** 10.3389/fgene.2026.1874407

**Published:** 2026-07-10

**Authors:** Marion Amujal, John Mukisa, Eric Katagirya, Samuel Kyobe, Thabo Diphoko, Savannah Mwesigwa, Gaseene Sebetso, Gerald Mboowa, Daudi Jjingo, David P. Kateete, Moses L. Joloba, Graeme Mardon, Neil A. Hanchard

**Affiliations:** 1 Department of Immunology and Molecular Biology, College of Health Sciences, Makerere University, Kampala, Uganda; 2 Childhood Complex Disease Genomics Section, National Human Genome Research Institute, NIH, Bethesda, MD, United States; 3 Department of Medical Microbiology, College of Health Sciences, Makerere University, Kampala, Uganda; 4 Department of Biological Sciences, University of Botswana, Gaborone, Botswana; 5 College of Computing and Information Sciences, Makerere University, Kampala, Uganda; 6 African Center of Excellence in Bioinformatics & Data Intensive Science, Infectious Diseases Institute, Kampala, Uganda; 7 Department of Pathology and Immunology, Baylor College of Medicine, Houston, TX, United States

**Keywords:** African genomic diversity, elite controllers, HIV host genetics, host pathogen interaction, long-term non-progressors, rare variant, viral control

## Abstract

The *CCR5* locus and the *HLA* class I region, first identified through candidate gene studies, remain the most well-characterized host genetic factors associated with HIV acquisition and disease progression, respectively. To date, genome wide association studies (GWAS) including those with several thousand individuals have largely replicated these same loci with very few additional associations identified. Increasingly, rare variants with larger effect sizes have been proposed to contribute to the unexplained inter-individual variability in HIV acquisition and disease progression. In this review, we highlight findings from rare variant studies across different populations and discuss the phenotypic and analytical challenges that impede discovery. We further highlight African ancestry cohorts as a valuable resource for rare variant discovery, given Africa’s extensive genetic diversity and disproportionate HIV burden. We emphasize the use of historical cohorts of persistently exposed but uninfected individuals as well as ART naïve HIV infected individuals, as these are increasingly difficult to recruit prospectively. Rare variant studies have the potential to reveal genes essential for HIV susceptibility and disease progression highlighting targets for novel HIV vaccines and therapeutics.

## 1 Introduction

HIV is still a major threat to global public health with 40.8 million people living with the virus in 2024, of these 1.3 million were new infections (UNAIDS, 2025). The burden is largely concentrated in Africa accounting for nearly two-thirds of all HIV infected individuals worldwide (UNAIDS, 2025). Over the last decade the expansion of antiretroviral therapy (ART) has contributed to remarkable clinical outcomes characterized by durable viral suppression and CD4^+^ T-cell recovery (Botey-Bataller et al., 2025; Fauci and Folkers, 2012). In 2024 nearly 77% of HIV infected individuals were receiving ART as opposed to 37% in 2014 (UNAIDS, 2025). However, HIV is still associated with high mortality with 630,000 AIDS related deaths reported in 2024 (UNAIDS, 2025).

HIV acquisition risk and subsequent clinical progression show significant interindividual variability. While socioeconomic and behavioral factors are important in determining acquisition risk (Dinçer et al., 2024), they do not fully explain the differences in HIV susceptibility. Some individuals remain uninfected despite multiple high-risk exposures to HIV, these are termed HIV exposed uninfected individuals (Horton et al., 2010; Kulkarni et al., 2003). Amongst individuals with established infection, a minority remain clinically and immunologically normal for sustained periods. These are the so-called elite controllers (ECs) (Deeks and Walker, 2007; Kayongo et al., 2018) and long-term non-progressors (LTNPs) (Zaunders and Van Bockel, 2013; Elahi et al., 2011). ECs spontaneously control the virus to extremely low levels (<50 copies/mL) without ART (Deeks and Walker, 2007; Kayongo et al., 2018), while LTNPs sustain low plasma viral loads (<10,000 copies/mL), high CD4^+^ T cell counts (>500 cells/µl), and remain ART naïve for over a decade (Zaunders and Van Bockel, 2013; Elahi et al., 2011; Capa et al., 2022). Although rare, these individuals are valuable natural models to understand mechanisms of durable viral control mediated by the host.

One of the major discoveries in HIV host genetics was the identification of a 32-base pair deletion in the CC-chemokine receptor 5 (*CCR5Δ32*) (Dean et al., 1996; Huang et al., 1996; Liu et al., 1996). This deletion yields a truncated receptor that blocks the entry of *CCR5* tropic HIV strains (Choe et al., 1996). Individuals homozygous for the deletion (*CCR5Δ32/Δ32*) show robust resistance to infection while heterozygous individuals progress more slowly to AIDS (Dean et al., 1996; Huang et al., 1996; Liu et al., 1996). These findings inspired the development of Maraviroc, a drug that blocks *CCR5* and prevents HIV entry (Fätkenheuer et al., 2005). The *CCR5Δ32* deletion has a frequency of ∼10% in European populations but is mostly absent elsewhere (Novembre et al., 2005). Candidate gene studies further revealed particular *HLA* class I alleles as determinants of HIV progression, notably HLA-B*57 and HLA-B*27 (Kaslow et al., 1996; Horton et al., 2006; Carrington and Walker, 2012). These alleles elicit potent cytotoxic T lymphocyte (CTL) responses that drive viral escape often at the cost of reduced viral fitness (Kelleher et al., 2001; Martinez-Picado et al., 2006; Schneidewind et al., 2007).

The advent of genome wide association studies (GWAS) introduced an era of hypothesis-free discovery (Hunter et al., 2008), yet in HIV host genetics these efforts have largely replicated the *CCR5* locus and the *HLA* class I region (McLaren et al., 2013; Lane et al., 2013; McLaren et al., 2015). A large GWAS of HIV acquisition risk involving 6,300 infected cases and 7,200 controls identified no associations beyond *CCR5Δ32* homozygosity and the rs4418214 SNP, which tags the protective HLA-B*57:01 and B*27:05 alleles. However, the association with rs4418214 was later shown to reflect frailty bias rather than a true effect on HIV acquisition (McLaren et al., 2013). Additionally, two studies found no evidence for *HLA* class I association with HIV acquisition in highly exposed uninfected individuals with hemophilia A or in sexually exposed uninfected individuals (Vince et al., 2014; Serrano-Rísquez et al., 2024), leaving *CCR5Δ32* homozygosity the only consistently replicated genetic determinant of HIV acquisition.

Beyond HIV susceptibility, GWAS of viral control and disease progression have identified only a limited number of associated loci. A large study of 6,315 individuals of European ancestry identified associations with HIV viral load control only at rs59440261 within the *HLA* region and at the intergenic variant rs1015164, which is in weak linkage disequilibrium with the *CCR5Δ32* deletion (McLaren et al., 2015). A separate functional study demonstrated that rs1015164 tags an ATF1 binding site that influences expression of the long non-coding RNA CCR5AS, which in turn regulates *CCR5* expression (Kulkarni et al., 2019). Notably, a GWAS of over 3,000 individuals of African ancestry identified a novel non-*HLA* variant, rs73001655 near *CHD1L* that was associated with a significant reduction in set-point viral load (spVL) (McLaren et al., 2023), this underscores the value of studying diverse populations. More recently, a multi-ancestry GWAS comprising 10,723 individuals implicated *ZNF586* as a novel regulator of HIV spVL. The limited number of GWAS findings relative to other complex traits suggests that additional genetic contributors including rare variants may play an important role (Trubetskoy et al., 2022; Xue et al., 2018; Meng et al., 2024).

Rare variants are not simply low frequency versions of common variants, they are shaped by distinct evolutionary pressures (Momozawa et al., 2021), and are defined as having a minor allele frequency (MAF) below 1%, with low frequency variants falling between 1% and 5% (Momozawa et al., 2021; Chen et al., 2022). They are evolutionarily young, originating from recent mutational events (Platt and Novembre, 2012), and exhibit reduced linkage disequilibrium with nearby loci (Momozawa et al., 2021). Rare variants are also more likely to be deleterious with purifying selection constraining them to low frequencies (Cirulli and Goldstein, 2010). The impact of these variants was first highlighted in the candidate gene study of *PCSK9*, where rare loss-of-function (LoF) variants were shown to markedly reduce low density lipoprotein (LDL) cholesterol and lower coronary heart disease risk (Cohen et al., 2006). These data enabled the development of monoclonal antibodies inhibiting *PCSK9* (Stein et al., 2012; Roth et al., 2012). This demonstrated that rare LoF variants can serve as natural human knockouts, providing targets for drug and vaccine development. In light of this, rare variants are increasingly being explored in HIV acquisition risk and disease progression. Here, we highlight findings from sequencing studies, discuss key challenges, and outline strategic directions for advancing rare variant studies.

## 2 Insights from rare variant studies in HIV acquisition and disease progression

### 2.1 Rare variant studies in European ancestry cohorts

An earlier multi-cohort study analyzed variants in protein coding regions, including rare variants, but did not yield any novel variant or gene associations. This study included over 1,300 Europeans from five HIV cohorts with spVL measurements. Gene-based association tests did not reveal any significant genes outside the major histocompatibility complex region (MHC) (McLaren et al., 2017). Further analysis in genes essential for HIV replication identified through siRNA knockdown (Zhu et al., 2014) and CRISPR screens (Park et al., 2017), also failed to uncover novel associations (McLaren et al., 2017). These results suggested that large effect coding genetic variation is unlikely to make a major contribution to HIV viral control. However, the absence of novel associations does not preclude a role for rare variants. It reveals the challenges of detecting associations in cohorts lacking sufficient sample sizes and genetic diversity. This therefore points out the need for sequencing in non-European populations as the next step in HIV host genetics.

**FIGURE 1:**
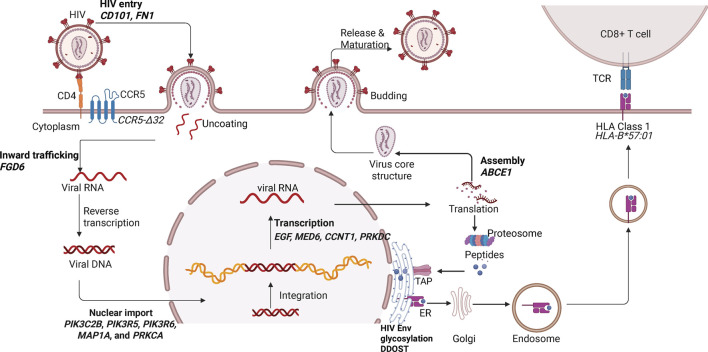
HIV replication cycle and the HLA class I mediated immune response. The HIV glycoprotein gp120 binds to the CD4 receptor and CCR5 co-receptor, triggering membrane fusion and release of the viral core into the cytoplasm. Single stranded viral RNA is then reverse transcribed into double stranded DNA. The formed pre-integration complex (PIC) is then transported to the nucleus where the viral DNA is integrated into the host genome. The integrated provirus is then transcribed into viral RNA, which is then exported to the cytoplasm and translated into viral polyproteins. Assembly of new virions then ensues, followed by budding and release of mature infectious particles. Host genes in bold indicate genes where rare variants modulate specific steps in the HIV replication cycle, including entry (*CD101*, *FN1*), inward trafficking (*FGD6*), nuclear import (*PIK3C2B*, *PIK3R5*, *PIK3R6*, *MAP1A*, *PRKCA*), transcription (*EGF*, *MED6*, *CCNT1*, *PRKDC*), HIV Env glycosylation (*DDOST*), and assembly (*ABCE1*). On the right, viral peptides are processed by the proteasome, transported via the transporter associated with antigen processing (TAP) to the endoplasmic reticulum. The resulting HLA-peptide complex is then exported through the Golgi apparatus to the surface of the cell, where it is presented to CD8^+^ T cells.

**FIGURE 2:**
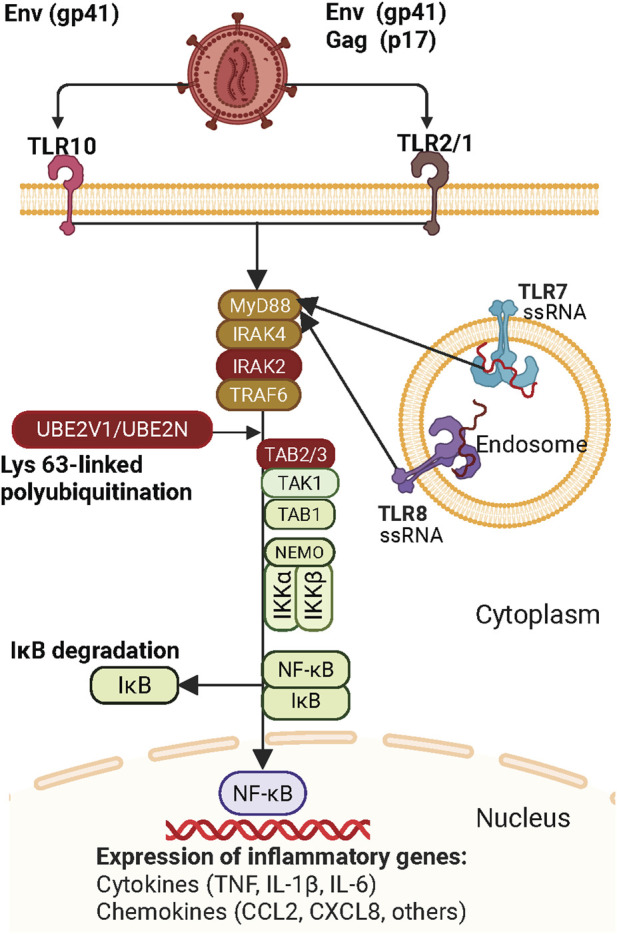
MyD88 dependent toll-like receptor (TLR) signaling pathway. TLR activation initiates assembly of the myddosome through recruitment of MyD88, IRAK4, and IRAK2. IRAK4 activates IRAK2 promoting TRAF6 recruitment. TRAF6 together with the UBE2V1-UBE2N complex catalyzes Lys63-linked polyubiquitination facilitating activation of the TAK1 kinase complex, which consists of TAK1, TAB1, TAB2, and TAB3. Activated TAK1 subsequently phosphorylates the IKK complex (NEMO, IKKα, IKKβ) leading to phosphorylation and degradation of IκB and release of NF-κB. NF-κB then translocates to the nucleus to induce transcription of pro-inflammatory cytokines. Rare variants in *IRAK2* (myddosome), *TAB2* (TAK1 complex), and *UBE2V1* (E2 ubiquitin-conjugating complex) have been associated with HIV acquisition and disease progression.

However, extending sequencing efforts to extreme phenotypes such as ECs and LTNPs has revealed additional genes associated with delayed AIDS onset (Nissen et al., 2018). In a study of European ancestry individuals from the Danish HIV Cohort, comprising seven untreated LTNPs and 4 ECs, strict filtering of 414,876 coding variants identified 24 rare variants across 20 genes. Most of these were exonic missense mutations with one stop-gain variant in *SLX4* and one splice-site variant in *PIK3R6*. Notably, no rare variants were found in three ECs, two of whom carried both the protective *CCR5Δ32* and HLA-B*57 alleles. The identified variants mapped to genes involved in distinct stages of the HIV lifecycle. These included genes implicated in viral entry (*FN1*), nuclear import (e.g., *PIK3C2B*, *PIK3R5*, *PIK3R6*), and HIV transcription (*EGF*, *MED6*, *CCNT1*, *PRKDC*) as detailed in Figure 1 and Table 1. Rare variants were further detected in genes implicated in innate immune sensing and inflammatory responses, including *IRAK2*, *LRRFIP1*, *TAB2*, *NOD2*, *SLX4* (Figure 2; Table 1). A rare variant was also found in *CMA1* implicated in SIV control (Nissen et al., 2018). Although limited by sample size, this analysis highlights genes that may contribute to HIV disease progression beyond the known protective loci.

**TABLE 1:** Summary of rare variant studies in HIV acquisition and disease progression across European and African ancestry populations.

Genes	HIV phenotype	Study population	Sequencing method	Major finding	Reference
*CD101* *UBE2V1*	HIV acquisition	African, 50 seroconverters and 50 HIV exposed seronegative	WGS	Rare variants in *CD101* and *UBE2V1* are associated with an increased risk of sexually acquired HIV	Mackelprang et al. (2017)
*FN1* *DDOST* *PIK3C2B* *PIK3R5* *PIK3R6* *MAP1A* *PRKCA* *FGD6* *MMP9* *FRK* *LRRFIP1* *IRAK2* *TAB2* *NOD2* *SLX4* *EGF* *MED6* *CCNT1* PRKDC *CMA1*	HIV progression	European, 7 LTNPs, 4 ECs, and 11 HIV-infected non-controllers	WES	Rare variants in multiple genes involved in immune signaling and HIV replication cycle contribute to HIV-1 control independent of *CCR5Δ32* and *HLA* alleles	Nissen et al. (2018)

### 2.2 Rare variant studies in African ancestry cohorts

Sequencing studies in African populations have proven particularly informative. Mackelprang et al. performed WGS in 100 adults (50 seroconverters and 50 seronegative individuals) recruited from HIV serodiscordant couples enrolled across three African cohorts: the Partners in Prevention HSV/HIV Transmission Study (Celum et al., 2010), the Couples Observational Study (Lingappa et al., 2011) and the Partners PrEP Study (Baeten et al., 2012). This study identified gene-level associations implicating *CD101* and *UBE2V1* in increased HIV acquisition risk (Figures 1, 2; Table 1) (Mackelprang et al., 2017). This was notable because they implicated genes not previously linked to HIV susceptibility in GWAS. Strongin et al. provided mechanistic evidence showing that CD101^+^ CD4^+^ T cells express disproportionately high levels of CCR5 and α4β7, rendering them highly susceptible to HIV/SIV infection (Strongin et al., 2022). Hence, CD101^+^ CD4^+^ T cells are preferentially depleted in the mucosal tissue and blood, a process linked to elevated viral loads and heightened inflammation (Strongin et al., 2022). In contrast, *UBE2V1* encodes an ubiquitin conjugating enzyme that facilitates TAK1 activation, promoting NF-κB dependent antiviral gene expression (Xia et al., 2009). *UBE2V1* further interacts with the known host restriction factor TRIM5-α, involved in HIV capsid uncoating (Pertel et al., 2011). These findings support a role for *CD101* and *UBE2V1* in susceptibility to HIV among African ancestry populations.

In addition to rare variant discovery, further analyses have examined whether HIV related genes show signatures of natural selection. One such study examined *ABCE1*, a host gene essential for HIV capsid assembly (Dooher et al., 2007). *ABCE1* resequencing in Yoruba individuals from Nigeria revealed a marked excess of rare variants, as depicted by a strongly negative Tajima’s D (−2.27) (Crawford et al., 2009). This excess of rare variants extended into the neighboring genes *ANAPC10* and *OTUD4* and was further observed in HIV positive African American individuals (Tajima’s D = −2.34) (Crawford et al., 2009). Although originally interpreted as evidence of positive selection, such patterns can also arise from demographic processes, including population expansion. However, it is unlikely that HIV driven selection pressure produced these signatures given the relatively recent emergence of the HIV/AIDS epidemic.

## 3 Analytical and phenotypic limitations in rare variant studies of HIV acquisition and disease progression

### 3.1 Sample size and statistical power limitations

Rare variant studies are limited by statistical power because power decreases as allele frequency decreases (Auer and Lettre, 2015). Variants with large effects may be observed in only a few individuals, leaving single variant association tests underpowered (Chen et al., 2022; Mathieson and McVean, 2012). To address this, methods that aggregate rare variants within genes or other biological units, such as burden and variance component tests are employed (Ionita-Laza et al., 2013). HIV progression studies also leverage individuals with extreme phenotypes such as LTNPs and ECs, while highly exposed but uninfected individuals, such as sex workers and serodiscordant couples, are prioritized for HIV acquisition studies (Horton et al., 2010; Pancino et al., 2010; Shearer and Clerici, 2010). These individuals are more likely to be enriched for variants of large effects. However, such cohorts are intrinsically small further limiting the ability to detect statistically robust associations.

### 3.2 Phenotype ascertainment challenges

Defining the contribution of rare variants in HIV acquisition and disease progression is limited by inaccurate phenotype ascertainment. In studies of sexually acquired HIV infection, exposure intensity depends on a variety of factors including the frequency of unprotected intercourse and the transmitting partner’s plasma viral load. Because the per-act transmission probability for vaginal intercourse is extremely low (∼1 in 1,250 exposures) (Hughes et al., 2012; Boily et al., 2009), even modest misclassification of exposure status can weaken associations. The retrospective design of most HIV progression studies also complicates phenotyping as undocumented ART use can lead to misclassification of individuals as LTNPs. Furthermore, cross sectional cohorts are subject to frailty bias. Individuals who survive long enough to be enrolled may be enriched for protective genotypes, thereby inflating effect size estimates (McLaren et al., 2013; Pressley and Patrick, 1999). Rare variant studies must therefore incorporate strict measures of phenotype ascertainment such that findings reflect the true underlying biology.

### 3.3 Representation gaps and inequities in genomic studies

The underrepresentation of African ancestry populations is one of the greatest barriers to rare variant discovery in HIV host genomics. African populations have the highest genetic diversity (Campbell and Tishkoff, 2008) yet sequencing efforts remain largely Eurocentric. Variant calling and filtering rely on population reference databases to separate functional rare variants from noise. However, owing to the insufficient representation of African genomes, allele frequency estimates are often unreliable and variants that are benign in African populations may be incorrectly flagged as pathogenic (Sirugo et al., 2019). Additionally, annotation tools trained on European biased datasets show reduced accuracy for African specific variants (Manrai et al., 2016), leaving functionally relevant variants incorrectly annotated.

The genetic diversity within the African continent itself add further complexity. With over 2,000 distinct ethnolinguistic groups, allele frequencies vary across populations, and admixture patterns are complex (Campbell and Tishkoff, 2008; Choudhury et al., 2020). This makes the definition of rare variants likely population specific. In addition, populations such as the southern African Khoe-San which represent one of the earliest diverging human lineages, remain sparsely represented in variant databases (Schlebusch et al., 2020; Retshabile et al., 2018). This limits discovery of variants unique to these ancient populations that may be relevant to HIV susceptibility and viral control. Ultimately, increased representation of African populations in genomic studies is needed to overcome these limitations.

### 3.4 Population stratification challenges

Rare variants are much more susceptible to population stratification and therefore more likely to lead to spurious associations (Mathieson and McVean, 2012). Population stratification is defined as the systematic difference in allele frequency across subpopulations due to restricted gene flow, geographic isolation, or nonrandom mating (Hellwege et al., 2017). Principal component analysis (PCA) (Price et al., 2006), genomic control (Devlin and Roeder, 1999) and mixed models (Kang et al., 2008) are widely used to correct for population stratification but were largely developed in the context of common variants. However, rare variants, owing to their recent origin and limited spread, tend to cluster within specific populations (Mathieson and McVean, 2012; Persyn et al., 2018). In a study that evaluated the performance of PCA for rare variant analyses by deriving the expected genetic relationship matrix as a function of allele frequency (Ma and Shi, 2020). The authors introduced two measures of population divergence: F_PC_, the ratio of inter-population variance to intra-population variance in the informative PCs, and d^2^, the sum of squared distances among populations (Ma and Shi, 2020). They demonstrate that population divergence metrics decrease with decreasing allele frequency indicating that PCA resolves population structure poorly for rare variants (Ma and Shi, 2020). These limitations underscore the need for methods specifically designed to correct for rare variant population stratification.

LocPerm addresses this challenge in studies with small sample sizes (Mullaert et al., 2021). It restricts phenotype permutations to genetically similar individuals, resolving a key limitation of standard permutation tests, which assume that all individuals are interchangeable regardless of ancestry (Mullaert et al., 2021). Among the methods tested, including standard permutation tests and principal component adjustment, LocPerm was the only method that maintained acceptable type I error control across all study conditions (Mullaert et al., 2021). This robustness at small sample sizes down to 30 cases, is directly relevant to rare HIV phenotypes such as LTNP and ECs.

### 3.5 Replication constraints

Replication of rare variant associations presents significant challenges mainly due to the low frequency and population specificity of these variants. In contrast to GWAS, where replication and the application of stringent significance thresholds (P < 5 × 10^−8^) are standard practice (Barsh et al., 2012; Huffman, 2018). Rare variant studies often suffer from a lack of appropriately matched replication cohorts. The major biobanks currently available are characterized by ancestry bias, for example, the United Kingdom Biobank consists predominantly of individuals of European ancestry (Sudlow et al., 2015), while Biobank Japan includes only Japanese participants (Nagai et al., 2017). In this case, alternative approaches such as functional studies may be necessary to confirm these associations.

### 3.6 Functional validation challenges

Identifying rare variants is only the first step, establishing their functional impact remains a major challenge. Primary CD4^+^ T cells which are the main targets of HIV are challenging to genetically manipulate (Schumann et al., 2015) and are subject to donor-to-donor variability (Longo et al., 2012). Immortalized cell lines, although easier to manipulate experimentally may fail to recapitulate the intrinsic cellular physiology necessary to model effects of rare variants (Pan et al., 2009). CRISPR based genome editing has improved the ability to functionally validate candidate variants by enabling the precise introduction of specific alleles into human T cells (Roth et al., 2018). This is more informative than overexpression experiments which may produce artefacts, for example, aberrant assembly of protein complexes at non-physiological expression levels (Ratz et al., 2015). However, precise knock-in via homology directed repair in primary CD4^+^ T cells remains technically challenging with reported efficiencies as low as 20% (Schumann et al., 2015). Alternatively, PBMCs from donors naturally carrying protective genotypes, when available, eliminate the need for genetic manipulation to functionally validate rare variant associations.

## 4 African genomic diversity as a resource for rare variant discovery in HIV acquisition and disease progression

As the geographic origin of modern humans, African populations are the most genetically diverse human populations with a complex demographic history and adaptation to diverse diets, climates and long-standing pathogen pressures (Campbell and Tishkoff, 2008). Initiatives such as the H3Africa Consortium have greatly advanced our understanding of African genomes. In one multi-cohort study, more than three million previously unreported genetic variants were identified, many of which were specific to underrepresented ethnolinguistic groups (Choudhury et al., 2020). In addition, 62 genomic regions under positive selection were detected including loci involved in antiviral immunity (Choudhury et al., 2020). This underscores the vast uncharacterized African genomic landscape and the strong imprint of pathogen pressures on human genetic diversity (Choudhury et al., 2020). For HIV host genetics studies, variants shaped by pathogen pressure are those most likely to influence acquisition and disease progression yet remain largely uncharacterized.

### 4.1 Pre-ART cohort as a valuable resource for rare variant studies of HIV disease progression

The widespread adoption of the universal test-and-treat policy while transformative for public health, has drastically reduced opportunities to study the natural history of HIV infection. Historical pre-ART cohorts therefore represent an invaluable resource. GWAS conducted in these cohorts have identified associations with HIV outcomes, including the *CHD1L* locus associated with HIV viral control (McLaren et al., 2023; Schulz et al., 2023). WES has further identified a novel allele HLA-C*03:02 associated with the LTNP phenotype in untreated pediatric cohorts (Kyobe et al., 2021). However, similar rare variant analyses in these populations remain scarce. Available pre-ART cohorts include the Children with HIV Early Antiretroviral Therapy (CHER) trial in South Africa (Violari et al., 2008; Cotton et al., 2013), cohorts enrolled through the International Maternal Pediatric Adolescent AIDS Clinical Trials (IMPAACT) network (Nachman, 2024), several cohorts established through routine clinical care (Soeria-Atmadja et al., 2020; Fitzgerald et al., 2019; Dondo et al., 2013), and those under the H3Africa consortium (Kyobe et al., 2023). The majority of them have archived specimens and detailed clinical histories documenting the period before treatment.

### 4.2 HIV exposed uninfected cohorts for rare variant discovery in HIV acquisition

In order to identify rare variants that influence HIV acquisition risk, studies must leverage cohorts of individuals who are highly exposed to HIV yet remain uninfected. Studies in African serodiscordant couples, including the Partners in Prevention HSV/HIV Transmission Study (Celum et al., 2010), the Couples Observational Study (Lingappa et al., 2011), and the Partners PrEP Study (Baeten et al., 2012), have already demonstrated the power of this approach, with rare variants in *UBE2V1* and *CD101* providing proof of concept (Mackelprang et al., 2017). Additional high-exposure populations with established cohorts include perinatally exposed but uninfected children enrolled through the IMPAACT network and the Zimbabwe Vitamin A for Mothers and Babies (ZVITAMBO) trial in Zimbabwe (Landes et al., 2012; Boivin et al., 2019; Evans et al., 2016). Other important cohorts include, female sex workers, such as those in the Pumwani cohort in Nairobi, Kenya (Lacap et al., 2008; Willim et al., 2022), men who have sex with men enrolled through the International AIDS Vaccine Initiative (IAVI) cohort in Eastern and Southern Africa (Price et al., 2021), and fisherfolk communities around Lake Victoria in Uganda (Ratmann et al., 2020). As pre-exposure prophylaxis (PrEP) coverage expands, individuals with repeated high risk exposures who remain infected will become increasingly difficult to recruit prospectively. Thus, sequencing of these archived biospecimens from HIV exposed yet uninfected individuals could facilitate further rare variant discovery.

## 5 Integrating host and viral genomics in HIV research

### 5.1 Viral genetic determinants of HIV control

Understanding HIV susceptibility and disease progression requires consideration of both host and viral genetic factors. Early evidence from rhesus macaques demonstrated that a deletion in the accessory gene *nef* reduced replication of simian immunodeficiency virus (SIV) and protected against progression to AIDS (Daniel et al., 1992). This was later observed in several studies in humans, including in the Sydney Blood Bank Cohort, where a donor and recipients infected with a strain of HIV with a deletion in nef and the nef/LTR overlap region had non-progressive HIV infection for over 10 years (Deacon et al., 1995; Learmont et al., 1999). Mutations in other viral genes have similarly been implicated in ECs and LTNPs, likely contributing to delayed disease progression (Alexander et al., 2000; Alexander et al., 2002). Additionally, host immune pressure also shapes viral evolution. In ECs and LTNPs, HLA-B*57 and HLA-B*58:01 restricted CD8^+^ T cells select for Gag escape mutations that reduce viral fitness (Minh et al., 2023; Miura et al., 2009). The *CHD1L* locus linked to reverse transcriptase and Gag escape mutations further supports a role for viral adaptation to host mediated immune pressure (McLaren et al., 2023; Schulz et al., 2023).

Furthermore, HIV subtype distribution also varies considerably by geography. Subtype C is the major strain in Southern Africa (Sivay et al., 2018), subtypes A and D in East Africa (Gounder et al., 2017; Bbosa et al., 2019), the circulating recombinant form CRF02_AG in West and Central Africa (Lihana et al., 2012; Nii-Trebi et al., 2017), while subtype B is mostly in North America, Europe and Australia (Tumiotto et al., 2018; Sallam et al., 2017; Taiaroa et al., 2024). Failing to account for this diversity risks confounding rare variant associations across cohorts.

### 5.2 Genome-to-genome analyses

Genome-to-genome (G2G) analyses simultaneously examine host and viral genomes to identify signatures of host-pathogen co-evolution. The first HIV G2G study conducted by Bartha *et al.*, analyzed paired host and viral data from 1,071 individuals and identified associations between host genetic variants and 48 HIV amino acid variants, all of which mapped to the *HLA* class I region (Bartha et al., 2013). These findings highlighted the role of *HLA* mediated pressure in shaping HIV sequence diversity.

Nonetheless, G2G studies in other viral infections have demonstrated the potential of this approach to uncover biologically meaningful non-*HLA* loci. For example, in hepatitis C virus infection, variation in *IFNL4* was shown to influence viral load through interaction with the viral NS5A polymorphism (Ansari et al., 2017). Applying G2G approaches to archived biospecimens from pre-ART African cohorts may therefore offer a promising avenue for uncovering novel host-virus interactions beyond *HLA*.

## 6 Discussion and conclusion

Earlier efforts to understand the host genetic determinants of HIV susceptibility and disease progression were led by candidate gene studies, starting with the identification of the *CCR5* and the *HLA* class I locus. Yet despite the promise of genome-wide hypothesis-free approaches, GWAS have mostly replicated the same loci. The scarcity of common variant associations compared with other complex traits, such as schizophrenia (Singh et al., 2017; Singh et al., 2022) and type 2 diabetes (Huerta-Chagoya et al., 2024), suggests that additional variant classes and more diverse populations should be explored.

Importantly, increasing evidence suggests that the genetic determinants of HIV susceptibility and disease progression are largely distinct (Serrano-Rísquez et al., 2024). Although some studies reported *HLA* associations with HIV acquisition risk (Luo, 2022), these findings have not been replicated in other highly exposed uninfected cohorts (Vince et al., 2014; Serrano-Rísquez et al., 2024). In hemophiliacs, this could be attributed to the high inoculum size delivered directly into the bloodstream, likely overwhelming *HLA*-mediated effects (Vince et al., 2014). However, the same lack of association is observed in sexually exposed uninfected individuals in whom the inoculum size is considerably smaller (Serrano-Rísquez et al., 2024). The extent to which this distinction extends to rare variants remains to be established.

An important consideration in rare variant studies is that although some large-effect rare variants may influence HIV susceptibility or viral control, their scarcity may reflect deleterious effects in other contexts. This is illustrated by *CCR5Δ32* homozygosity, which provides complete protection against *CCR5* tropic HIV, but increases the risk of symptomatic West Nile virus infection (Glass et al., 2006). Rare variants may carry similar pleiotropic effects that are not evident from HIV studies alone, this should be considered when prioritizing candidates for follow-up.

Realizing the full potential of rare variants will require a number of key strategies, including larger sample sizes enriched for African ancestry, achieved through the meta-analysis of multicohort data, pre-ART historical cohorts, as well as highly exposed but uninfected individuals. Among these, children who are perinatally exposed but uninfected represent a particularly valuable resource. Unlike adults, where exposure intensity depends on self-reported behavioral data, exposure in children can be inferred from maternal viral load (Mock et al., 1999), enabling more precise phenotype ascertainment. However, when infection occurs, ART-naïve pediatric non-progressors offer a natural model of viral control with a clearly defined infection period, further improving the accuracy of phenotype classification. To support these studies, the existing infrastructure and expertise from H3Africa funded initiatives (Mlotshwa et al., 2017; Mboowa et al., 2018) and the H3Africa Biorepositories (Nsubuga et al., 2022) should be leveraged. Ethics must be taken into consideration with clear data sharing and transparent informed consent processes. This will require support from governments, academic institutions and international partners.

Furthermore, with the rapid expansion of long-read sequencing technologies (Rhoads and Au, 2015; Lin et al., 2021), rare variant studies must extend beyond coding variation to capture additional classes of genetic variants. These include copy number variants such as *CCL4L* and *CCL3L*, which have previously been implicated in HIV susceptibility (Shostakovich-Koretskaya et al., 2009), as well as inversions, microsatellites, repeat expansions, and transposon insertions, particularly human endogenous retroviruses. Exome sequencing also fails to detect cryptic intronic variants that may disrupt splicing of genes with potential roles in HIV infection. Addressing these limitations will further require integrating WGS data with RNA sequencing to identify variants that influence gene expression. These advances will ultimately enhance rare variant discovery and deepen our understanding of the mechanisms underlying HIV susceptibility and viral control, potentially guiding the development of novel vaccines and therapeutics.
